# Analytical wave solutions in thermoelastic media with temperature-dependent properties via IMETF method

**DOI:** 10.1038/s41598-025-09344-w

**Published:** 2025-09-15

**Authors:** Mohamed F. Ismail, Hamdy M. Ahmed, Soha Safwat, M. Elsaid Ramadan, Nesreen Sirelkhtam Elmki Abdalla, Mahmoud Soliman

**Affiliations:** 1 Faculty of Computers and Information System, Egyptian Chinese University, Cairo, Egypt; 2 Department of Physics and Engineering Mathematics, Higher Institute of Engineering, El Shorouk Academy, Cairo, Egypt; 3 Department of Mathematics, Faculty of Science, Islamic University of Madinah, Medina, Saudi Arabia; 4 Department of Mathematics, College of Science, King Khalid University, Abha, Saudi Arabia; 5 Department of Physics and Engineering Mathematics, Faculty of Engineering, Ain Shams University, Cairo, Egypt

**Keywords:** Three-phase-lag model, Nonlinear thermoelasticity, Temperature-dependent, IMETF technique, Exact wave solutions, Applied mathematics, Computational science

## Abstract

This study delivers an in-depth analytical investigation of exact wave solutions derived within the context of the Three-Phase-Lag (3PHL) generalized thermoelasticity model, explicitly incorporating the temperature dependence of material properties. By applying the Improved Modified Extended Tanh Function (IMETF) method, the research addresses the governing equations that describe the coupled interaction between thermal and mechanical fields in solids. A central feature of this work is the inclusion of temperature-sensitive material parameters, which play a crucial role in modifying thermoelastic responses under a variety of thermal and mechanical loading scenarios. Unlike traditional approaches, the IMETF method extends the classical tanh-function technique by introducing a more flexible solution structure capable of capturing a richer set of waveforms. This improved methodology facilitates the derivation of diverse exact analytical solutions, each governed by distinct free parameters. These include hyperbolic, singular hyperbolic, exponential, Weierstrass elliptic, and bell-shaped solitary wave solutions. Each solution class offers unique physical insights into wave propagation behavior within temperature-dependent thermoelastic media. The analytical results not only deepen the theoretical understanding but also uncover critical features of wave interaction, dispersion, and attenuation in materials governed by the 3PHL model. To further support and illustrate these findings, the paper includes detailed graphical visualizations of key physical quantities such as stress tensor components, displacement fields, and temperature distributions. These visual results serve to highlight the influence of the temperature dependence on the wave dynamics.

## Introduction

Thermoelasticity under the 3PHL model represents a significant advance in the theoretical and applied analysis of heat conduction and deformation in solids subjected to thermal loads. Unlike the classical Fourier law, which assumes an infinite speed of heat propagation, the 3PHL model introduces three distinct time delays–or phase lags–associated with the heat flux vector, temperature gradient, and strain field. This allows for a more realistic and refined description of thermal interactions in micro- and nano-scale systems or materials experiencing high-frequency thermal processes. The three lags, typically denoted as $\tau _{q}$ (heat flux delay), $\tau _{\theta }$ (temperature gradient delay) and $\tau _{\nu }$ (strain delay), enable the model to account for non-Fourier heat conduction behaviors and the coupling between thermal and mechanical responses more accurately than the Dual-Phase-Lag (DPL) model or earlier theories such as those by Lord–Shulman and Green–Lindsay. When incorporated into the framework of generalized thermoelasticity, the 3PHL theory leads to a system of hyperbolic Partial Differential Equations (PDEs), which describe the wave-like nature of heat propagation and the resulting thermoelastic disturbances. The inclusion of these three lags is particularly relevant for the study of materials with complex internal structures, functionally graded media, temperature-dependent properties, or those subjected to ultrafast laser pulses. Analytical and numerical investigations under this model have demonstrated notable differences in temperature and displacement fields, for more details see^1–10^. Several recent studies have explored advanced thermoelastic and viscoelastic models to capture complex physical phenomena such as damping, wave propagation, and memory effects in various media. For example, the work^11^ presents a thorough investigation of wave behavior under different thermoelastic theories, providing insight into how magnetic fields and conductivity influence stress waves across the classical and Green-Lindsay frameworks. In parallel, Al-Jamel et al.^12^ introduces a novel fractional-order approach to describe damping with memory effects, which is particularly relevant for materials exhibiting history-dependent behavior. Similarly, the study^13^ extends fractional calculus techniques to viscoelastic fluid dynamics, providing a deeper understanding of how hereditary effects shape the response under transient thermal loads. Further exploring biological applications, Tiwari^14^ applies a fractional framework to capture the intricate thermal behavior in soft tissues, highlighting the significance of non-local and non-singular kernels in modeling bio-heat transfer. In work^15^ investigates nanoscale damping mechanisms by extending classical models to incorporate nonlocal elasticity and thermoelastic coupling, shedding light on how scale effects impact energy dissipation in micro/nano structures. Collectively, these works contribute to the evolving field of generalized thermoelasticity and fractional mechanics, demonstrating the importance of nonlocal, memory-dependent, and size-sensitive modeling in modern materials science. Furthermore, the 3PHL model provides a better theoretical basis for designing and analyzing micro-electro-mechanical systems, biomedical devices, and advanced composite materials, where the interaction between heat and deformation must be accurately captured for improved performance and reliability. Thus, the study of thermoelasticity within the 3PHL framework opens new avenues for addressing cutting-edge engineering problems and contributes to the broader understanding of coupled thermal-mechanical processes in modern materials.

Incorporating the effects of temperature-dependent properties into thermoelastic models is crucial for achieving realistic simulations of how materials behave under combined thermal and mechanical influences in a wide range of engineering applications. Traditional theories of thermoelasticity often assume that material parameters such as thermal conductivity, thermal expansion coefficients, and the Lamé constants, remain constant. However, numerous experimental investigations have shown that these properties are in fact sensitive to temperature variations, particularly in high-temperature settings commonly encountered in sectors such as aerospace, nuclear energy systems, and microelectronic fabrication^16^. To better represent actual material responses, modern theoretical frameworks in thermoelasticity have evolved to include temperature-dependent material characteristics, which inherently introduce nonlinearity into the governing field equations. These nonlinear formulations are essential for accurately capturing the way materials react to thermal loads, especially in environments where significant thermal gradients and mechanical stresses coexist. As the temperature rises, materials may undergo notable changes in physical behavior–such as thermal softening or stiffening–which directly influence stress wave propagation, energy absorption, and internal stress distributions^17^. This temperature responsiveness is particularly significant in transient heat conduction problems, where rapid and localized temperature shifts may lead to concentrated thermal stresses^10,18^. To accommodate such complexities, extended versions of generalized thermoelastic theories–including modified forms of the Lord–Shulman and Green–Naghdi models–have been reformulated to incorporate variable coefficients that evolve with temperature. These theoretical refinements offer a more precise platform for modeling thermomechanical behavior in advanced materials. However, the introduction of temperature-dependent properties considerably complicates the mathematical structure of the system, often requiring the use of sophisticated analytical tools and numerical approaches to derive meaningful solutions. To manage the intricate challenges inherent in thermoelastic systems, researchers have proposed a variety of mathematical and computational strategies. Among these, sophisticated numerical simulation techniques^19,20^ and analytical approaches like perturbation methods^21,22^ have played a pivotal role in delivering accurate predictions and insights. In particular, recent work by Ismail et al. in 2025^23,24^ marked a significant advance by presenting an exact analytical solution, offering deeper insight into the complex coupling between thermal and mechanical effects. Investigations into thermoelastic models that consider temperature-dependent material properties have revealed distinct characteristics in wave behavior, such as dissipation and dispersion phenomena^25,26^. In recent years, numerous researchers have investigated various physical effects on thermoelastic responses under different theoretical models^27–38^. Consequently, accurately modeling temperature-dependent behavior is now regarded as a fundamental requirement in the development and performance assessment of high-performance structural materials, particularly those designed to withstand extreme thermal conditions while maintaining mechanical integrity, such as next-generation composites and thermal protection systems.

Exact analytical solutions hold a fundamental position in the study of complex physical systems, as they precisely satisfy the governing equations without relying on approximations. This makes them invaluable for uncovering the underlying mechanisms of nonlinear processes and for offering transparent interpretations of intricate wave behaviors. One particularly important class of such solutions in nonlinear science is solitons, stable localized waveforms that preserve their shape and velocity during propagation. Their existence arises from a finely tuned balance between nonlinear effects and dispersion within the medium. The exploration of exact solutions, especially soliton-type solutions, significantly advances our comprehension of various scientific and engineering domains, including plasma dynamics, hydrodynamics, and the transmission of signals in optical fibers^39–45^. To derive such solutions, researchers have developed a diverse array of sophisticated mathematical frameworks. Among these, the Hirota bilinear method stands out as a widely adopted technique for generating multi-soliton solutions^46–48^, which has been instrumental in studying the interactions, collisions, and long-term stability of solitary waves. In addition, the inverse scattering transform remains one of the most powerful analytical tools for solving integrable Non Linear Partial Differential Equations (NLPDEs)^49,50^, providing deep insight into the evolution and characteristics of solitonic structures in various physical contexts. Beyond their theoretical value, exact solutions are also crucial for practical applications–they act as reliable benchmarks for validating numerical schemes and experimental results, thus enhancing the robustness and accuracy of computational models used to simulate nonlinear wave propagation. In general, the pursuit and application of exact solutions enrich both the analytical and applied understanding of nonlinear phenomena in natural and engineered systems.

The IMETF method is a powerful and versatile analytical tool designed to derive exact solutions for NLPDEs that frequently arise in a wide array of scientific and engineering contexts. This method builds upon and significantly enhances the classical tanh-function approach by introducing additional parameters and structural extensions, thereby increasing its adaptability and effectiveness in addressing a broader spectrum of nonlinear problems. Central to the IMETF technique is the application of an appropriate wave transformation, which simplifies the original NLPDEs into a more manageable Ordinary Differential Equation (ODE). This transformation paves the way for the systematic construction of exact solutions that can exhibit diverse wave profiles, including soliton-like forms, exponential wave patterns, and other intricate solution structures. The robustness and flexibility of the IMETF method have made it particularly useful in tackling advanced mathematical models in areas such as generalized thermoelasticity and nonlinear wave propagation in optical fiber systems^51–55^. Through its ability to reveal explicit analytical forms, the method offers significant insight into the dynamics of wave behavior in these complex systems. Moreover, the solutions obtained not only deepen theoretical understanding but also contribute to the accurate characterization of physical phenomena such as stress wave interaction, thermal diffusion, and signal transmission. Consequently, the IMETF approach serves as an essential analytical framework for exploring and interpreting nonlinear wave mechanics across various physical and technological applications.

The primary objective of this study is to conduct a comprehensive and rigorous analysis of how temperature dependence influences the behavior of thermoelastic materials. This exploration is carried out within the framework of the 3PHL model, utilizing the IMETF technique as the central analytical tool. In the subsequent section, we provide an in-depth overview of the IMETF method, emphasizing its theoretical foundation, importance, and effectiveness in solving NLPDEs. Building upon this methodological base, the study proceeds to investigate the critical effects of temperature variability on the dynamic and structural responses of thermoelastic systems. Special attention is devoted to understanding how fluctuations in temperature can markedly alter the material response, energy distribution, and wave propagation characteristics across different thermoelastic media. To enrich the analysis, the research presents a diverse spectrum of exact solutions, encompassing hyperbolic, sigular hyperbolic, exponential, Weierstrass elliptic, and bell shaped solitary solutions. These solutions not only illuminate the range of possible behaviors exhibited by thermoelastic systems under varying thermal conditions but also contribute to the broader understanding of wave phenomena in nonlinear media. To further enhance the interpretability of the results, the study incorporates 2D graphical representations, which serve to visually convey the key features, trends, and outcomes observed in the analysis. These visualizations offer intuitive insights into the temperature-dependent behavior of thermoelastic materials, supporting the theoretical findings and highlighting their potential applications in engineering and materials science.

## The IMETF technique summary

The IMETF technique is introduced in this section^56,57^.

Taking into account the subsequent NLPDE:

1 $$\begin{aligned} F(u,u_\varsigma ,u_x,u_{xx},u_{\varsigma x},....)=0, \end{aligned}$$

where *F* represents the equivalent partial derivatives for space and time of the polynomial function $u(x,\varsigma )$.

The IMETF method is an effective analytical technique for deriving approximate solutions to NLPDEs. This approach begins by transforming the original dependent variable *u* into a new variable *A* through an appropriate transformation function. After the transformation, the equation is simplified by discarding higher-order terms and making suitable approximations.

Using this method allows one to obtain approximate analytical solutions to the NLPDE, offering significant insights into the system’s underlying dynamics and behavior.

For additional information and examples regarding the IMETF technique , consult reference^56^.

To solve Eq. (1) using this methodology, the following steps should be undertaken:

*Step-1*: Begin by applying a suitable transformation to reduce Eq. (1) into an ODE:

2 $$\begin{aligned} u(x,\varsigma ) = A(\epsilon ), \quad \epsilon = x - \nu \varsigma , \end{aligned}$$

where $\nu$ represents the wave velocity associated with wave propagation. As a result, the NLPDE given in Eq. (1) is transformed into the following form:

3 $$R(A,A^{\prime } ,A^{{\prime \prime }} ,A^{{\prime \prime \prime }} , \ldots ) = 0.$$

*Step-2*: The solution to Eq. (3) is assumed in the form:

4 $$\begin{aligned} A(\epsilon ) = \sum _{j=0}^{N} a_j \Upsilon ^j(\epsilon ) + \sum _{j=1}^{N} b_j \Upsilon ^{-j}(\epsilon ), \end{aligned}$$

where $\Upsilon (\epsilon )$ meets the following auxiliary equation:

5 $$\begin{aligned} \Upsilon '(\epsilon ) = \sqrt{d_0 + d_1 \Upsilon (\epsilon ) + d_2 \Upsilon ^2(\epsilon ) + d_3 \Upsilon ^3(\epsilon ) + d_4 \Upsilon ^4(\epsilon )}. \end{aligned}$$

*Step-3*: Determine the positive number *N* in Eq. (4) by employing the balancing method on Eq. (3).

*Step-4*: Insert the differential equation from Eq. (5) and the proposed solution form into Eq. (3). Then, set the coefficients of each power of $\Upsilon ^p(\xi )$ to zero for $p = 0, \pm 1, \pm 2, \dots$, leading to a system of nonlinear algebraic equations.

*Step-5*: Solve the system derived in Step-4 using Mathematica to find the unknowns $a_j$, $b_j$, and $\nu$.

*Step-6*: Different choices for the constants $d_0$, $d_1$, $d_2$, $d_3$, and $d_4$ yield a variety of solution types.

**Case 1:** $$d_0=d_1=d_3=0$$

$$\begin{aligned} \Upsilon (\epsilon )=\sqrt{-\frac{d_2}{d_4}} \text {sech}(\sqrt{d_2} \epsilon ),\quad d_2>0, d_4<0. \end{aligned}$$

**Case 2:** $$d_2=d_4=0,$$

$$\begin{aligned} \Upsilon (\epsilon )=\wp \left( \dfrac{\sqrt{d_3}}{2} \epsilon ,\dfrac{-4d_1}{d_3},\dfrac{-4d_0}{d_3}\right) \quad d_0\ne 0, d_1\ne 0 , \end{aligned}$$

**Case 3:** $$d_{0}=d_1=d_{2}=0$$

$$\begin{aligned} \Upsilon (\epsilon ) = \dfrac{d_3 \exp \left( \dfrac{d_3 \epsilon }{2\sqrt{-d_4}}\right) }{2d_4}\quad d_{4}<0, \end{aligned}$$

**Case 4:** $$d_0=d_1=d_4=0$$

$$\begin{aligned} \Upsilon (\epsilon )=-\frac{d_2 \text {sech}^2\left( \frac{1}{2} \sqrt{d_2} \epsilon \right) }{d_3},\quad d_2>0. \end{aligned}$$

**Case 5:**$$d_3=d_4=d_0=0$$

$$\begin{aligned} \Upsilon (\epsilon ) =\frac{-d_1}{2d_2} +\sqrt{\frac{d_{1}}{2d_{2}}} \sinh (2\sqrt{d_{2}}\epsilon )\quad d_{2}>0, \end{aligned}$$

*Step-7*: Numerous solutions to Eq. (1) can be obtained by substituting the determined constants $a_j$, $b_{j}$, along with the previously derived general solutions of Eq. (5), into Eq. (4).

## Basic equations

This section explores the effect of temperature dependence on thermoelastic behavior within the specified range $0\preceq z\le \infty$.

The following equations define the displacement components:

$$\begin{aligned} w=\left( z,\varsigma \right) ,\;u=v=0. \end{aligned}$$

Therefore, the motion equation in one-dimensional form is represented as follows:^58^:

6 $$\begin{aligned} \sigma _{,z}=\rho w_{,\varsigma \varsigma }, \end{aligned}$$

and the stress tensor is expressed by:

7 $$\begin{aligned} \sigma =\left( \lambda +2\mu \right) w_{,z}-\gamma T. \end{aligned}$$

The one-dimensional heat conduction equation within the 3PHL model is now given as follows:

8 $$\begin{aligned} \left( 1+\tau _{\theta }\frac{\partial }{\partial \varsigma }\right) \left( kT_{,z\varsigma }\right) _{,z}+\left( 1+\tau _{\nu }\frac{\partial }{\partial \varsigma } \right) \left( k^{*}T_{,z}\right) _{,z}=\left( 1+\tau _{q}\frac{\partial }{\partial \varsigma }+\frac{\tau _{q}^{2}}{2}\frac{\partial ^{2}}{\partial \varsigma ^{2}} \right) \left[ \rho c_{e}T_{,\varsigma \varsigma }+\gamma T_{0}w_{,z\varsigma \varsigma }\right] , \end{aligned}$$

where *T* is such that $\vert \frac{ T}{T_{0}}\vert \prec \prec 1,$ $0\prec \tau _{\nu } \prec \tau _{\theta } \prec \tau _{q}$, $0\prec k$ and $0\prec k^{*}$

For temperature-dependent materials, we assume that

9 $$\begin{aligned} \left( \lambda ,\mu ,\gamma ,\rho ,k, k^{*}\right) =\left( \lambda _{0},\mu _{0}, \gamma _{0}, \rho _{0}, k_{0}, k_{0}^{*}\right) f\left( T\right) . \end{aligned}$$

In this scenario, let $f\left( T\right)$ is a continuous within the range $0\preceq T \prec \infty$ while $\lambda _{0},$ $\mu _{0},$ $\gamma _{0},$ $\rho _{0},$ $k_{0}$, and $k_{0}^{*}$ are constants.

Inserting Eq. (9) into Eqs. (6)–(8), we find

10 $$\begin{aligned} \sigma _{,z}=\rho _{0}f\left( T\right) w_{,\varsigma \varsigma }, \end{aligned}$$

11 $$\begin{aligned} \sigma =f\left( T\right) \left[ \left( \lambda _{0}+2\mu _{0}\right) w_{,z}-\gamma _{0}T\right] , \end{aligned}$$

12 $$\begin{aligned} & \left( 1+\tau _{\theta }\frac{\partial }{\partial \varsigma }\right) \left( k_{0}f\left( T\right) T_{,z\varsigma }\right) _{,z}+\left( 1+\tau _{\nu }\frac{ \partial }{\partial \varsigma }\right) \left( k_{0}^{*}f\left( T\right) T_{,z}\right) _{,z}\nonumber \\ & \quad =\left( 1+\tau _{q}\frac{\partial }{\partial \varsigma }+\frac{ \tau _{q}^{2}}{2}\frac{\partial ^{2}}{\partial \varsigma ^{2}}\right) \left[ \rho _{0}f\left( T\right) c_{e}T_{,\varsigma \varsigma }+\gamma _{0}f\left( T\right) T_{0}w_{,z\varsigma \varsigma } \right] . \end{aligned}$$

The following nondimensional variables are utilized

13 $$\begin{aligned} \left( \tilde{z}, \tilde{w}\right) =\omega c_{0}\left( z, w\right) , \left( \tilde{\varsigma }, \tilde{\tau }_{\theta }, \tilde{\tau }_{\nu }, \tilde{\tau }_{q}\right) =c_{0}^{2}\omega \left( \varsigma , \tau _{\theta }, \tau _{\nu }, \tau _{q}\right) , \tilde{T}=\frac{T}{T_{0}}, \tilde{\sigma }=\frac{\sigma }{ \lambda _{0}+2\mu _{0}}, \end{aligned}$$

where $$c_{0}^{2}=\frac{\lambda _{0}+2\mu _{0}}{\rho _{0}}$$ and $$\omega = \frac{\rho _{0}c_{e}}{k_{0}}.$$

Using Eq. (13) in Eq. (10) with the aid of Eq. (11), one infers

14 $$\begin{aligned} f\left( \tilde{T}\right) \left[ \tilde{w}_{,\tilde{z}\tilde{z}}-a_{1}\tilde{T }_{,\tilde{z}}-\tilde{w}_{,\tilde{\varsigma }\tilde{\varsigma }}\right] =f^{^{\prime }}\left( \tilde{T}\right) \tilde{T}_{,\tilde{z}}\left[ \tilde{w}_{,\tilde{z}}-a_{1} \tilde{T}\right] , \end{aligned}$$

where $$f^{^{\prime }}\left( \tilde{T}\right) =\frac{df\left( \tilde{T} \right) }{d\tilde{T}}$$ and $$a_{1}=\frac{T_{0}\gamma _{0}}{2\mu _{0}+\lambda _{0}}$$.

When Eq. (13) is used in Eq. (12), it follows that

15 $$\begin{aligned} 0= & f\left( \tilde{T}\right) \{\tilde{\tau }_{\theta }\tilde{T}_{,\tilde{z} \tilde{z}\tilde{\varsigma }\tilde{\varsigma }}+a_{2}\tilde{T}_{,\tilde{z}\tilde{z}}+a_{3} \tilde{T}_{,\tilde{z}\tilde{z}\tilde{\varsigma }}-a_{4}\tilde{T}_{,\tilde{\varsigma }\tilde{\varsigma } }-a_{5}\tilde{w}_{,\tilde{z}\tilde{\varsigma }\tilde{\varsigma }}-a_{6}\tilde{T}_{,\tilde{\varsigma } \tilde{\varsigma }\tilde{\varsigma }}-a_{7}\tilde{w}_{,\tilde{z}\tilde{\varsigma }\tilde{\varsigma }\tilde{\varsigma } }-a_{8}\tilde{T}_{,\tilde{\varsigma }\tilde{\varsigma }\tilde{\varsigma }\tilde{\varsigma }} \nonumber \\ & -a_{9}\tilde{w}_{,\tilde{z}\tilde{\varsigma }\tilde{\varsigma }\tilde{\varsigma }\tilde{\varsigma } }\}+f^{^{\prime }}\left( \tilde{T}\right) \{a_{10}\tilde{T}_{,\tilde{z}} \tilde{T}_{,\tilde{z}\tilde{\varsigma }}+\tilde{\tau }_{\theta }\left( \tilde{T}_{, \tilde{z}\tilde{\varsigma }}\right) ^{2}+\tilde{\tau }_{\theta }\tilde{T}_{,\tilde{\varsigma }} \tilde{T}_{,\tilde{z}\tilde{z}\tilde{\varsigma }}+\tilde{\tau }_{\theta }\tilde{T}_{, \tilde{z}}\tilde{T}_{,\tilde{z}\tilde{\varsigma }\tilde{\varsigma }}+a_{2}\left( \tilde{T}_{, \tilde{z}}\right) ^{2} \nonumber \\ & +a_{11}\tilde{T}_{,\tilde{\varsigma }}\tilde{T}_{,\tilde{z}\tilde{z}}-a_{6}\tilde{T} _{,\tilde{\varsigma }}\tilde{T}_{,\tilde{\varsigma }\tilde{\varsigma }}-a_{7}\tilde{T}_{,\tilde{\varsigma }} \tilde{w}_{,\tilde{z}\tilde{\varsigma }\tilde{\varsigma }}-a_{8}\left( \tilde{T}_{,\tilde{\varsigma } \tilde{\varsigma }}\right) ^{2}-a_{9}\tilde{T}_{,\tilde{\varsigma }\tilde{\varsigma }}\tilde{w}_{, \tilde{z}\tilde{\varsigma }\tilde{\varsigma }}\}+f^{^{\prime \prime }}\left( \tilde{T}\right) \{\tilde{\tau }_{\theta }\tilde{T}_{,\tilde{\varsigma }}\tilde{T}_{,\tilde{z}}\tilde{T} _{,\tilde{z}\tilde{\varsigma }} \nonumber \\ & +a_{11}\tilde{T}_{,\tilde{\varsigma }}\left( \tilde{T}_{,\tilde{z}}\right) ^{2}-a_{8}\left( \tilde{T}_{,\tilde{\varsigma }}\right) ^{2}\tilde{T}_{,\tilde{\varsigma } \tilde{\varsigma }}-a_{9}\left( \tilde{T}_{,\tilde{\varsigma }}\right) ^{2}\tilde{w}_{,\tilde{z }\tilde{\varsigma }\tilde{\varsigma }}\}, \end{aligned}$$

where $$f^{^{\prime \prime }}\left( \tilde{T}\right) =\frac{d^{2}f\left( \tilde{T}\right) }{d\tilde{T}^{2}},$$ $$a_{2}=\frac{k_{0}^{*}}{ k_{0}c_{0}^{2}\omega },$$ $$a_{3}=1+\tilde{\tau }_{\nu }a_{2},$$ $$a_{4}=\frac{ c_{e}\rho _{0}}{k_{0}\omega },$$ $$a_{5}=\frac{\gamma _{0}}{k_{0}\omega },$$ $$a_{6}=\tilde{\tau }_{q}a_{4},$$ $$a_{7}=\tilde{\tau }_{q}a_{5},$$

$$a_{8}=\frac{\tilde{\tau }_{q}^{2}}{2}a_{4},$$ $$a_{9}=\frac{\tilde{\tau } _{q}^{2}}{2}a_{5},$$ $$a_{10}=1+2\tilde{\tau }_{\nu }a_{2},$$ $$a_{11}=\tilde{\tau }_{\nu }a_{2}.$$

Using Eq. (13) in Eq. (11), we see

16 $$\begin{aligned} \tilde{\sigma }=f\left( \tilde{T}\right) \left[ \tilde{w}_{,\tilde{z}}-a_{1} \tilde{T}\right] . \end{aligned}$$

The subsequent function is adopted from^24^:

$$\begin{aligned} f\left( T\right) =1-\frac{\alpha }{T_{0}}T, \end{aligned}$$

where $\alpha$ is the temperature-sensitivity parameter. This form is commonly used in the literature for moderate temperature variations, as it allows for analytical tractability while capturing first-order thermal effects on material properties. .

The following expression is deduced through the use of the dimensionless variable:

17 $$\begin{aligned} f\left( \tilde{T}\right) =1-\alpha \tilde{T}. \end{aligned}$$

From Eq. (17) in Eqs. (14), (15), and (16), one acquires

18 $$\begin{aligned} \tilde{w}_{,\tilde{z}\tilde{z}}-\alpha \tilde{T}\tilde{w}_{,\tilde{z}\tilde{z }}-\tilde{w}_{,\tilde{\varsigma }\tilde{\varsigma }}-a_{1}\tilde{T}_{,\tilde{z}}+\alpha \tilde{ T}\tilde{w}_{,\tilde{\varsigma }\tilde{\varsigma }}+\alpha \tilde{T}_{,\tilde{z}}\tilde{w}_{, \tilde{z}}=0, \end{aligned}$$

19 $$\begin{aligned} 0= & \tilde{\tau }_{\theta }\tilde{T}_{,\tilde{z}\tilde{z}\tilde{\varsigma }\tilde{\varsigma } }+a_{2}\tilde{T}_{,\tilde{z}\tilde{z}}+a_{3}\tilde{T}_{,\tilde{z}\tilde{z} \tilde{\varsigma }}-a_{4}\tilde{T}_{,\tilde{\varsigma }\tilde{\varsigma }}-a_{5}\tilde{w}_{,\tilde{z} \tilde{\varsigma }\tilde{\varsigma }}-a_{6}\tilde{T}_{,\tilde{\varsigma }\tilde{\varsigma }\tilde{\varsigma }}-a_{7} \tilde{w}_{,\tilde{z}\tilde{\varsigma }\tilde{\varsigma }\tilde{\varsigma }}-a_{8}\tilde{T}_{,\tilde{\varsigma } \tilde{\varsigma }\tilde{\varsigma }\tilde{\varsigma }} \nonumber \\ & -a_{9}\tilde{w}_{,\tilde{z}\tilde{\varsigma }\tilde{\varsigma }\tilde{\varsigma }\tilde{\varsigma }}-\alpha \tilde{\tau }_{\theta }\tilde{T}\tilde{T}_{,\tilde{z}\tilde{z}\tilde{\varsigma }\tilde{ t}}-\alpha a_{2}\tilde{T}\tilde{T}_{,\tilde{z}\tilde{z}}-\alpha a_{3}\tilde{T }\tilde{T}_{,\tilde{z}\tilde{z}\tilde{\varsigma }}+\alpha a_{4}\tilde{T}\tilde{T}_{, \tilde{\varsigma }\tilde{\varsigma }}+\alpha a_{5}\tilde{T}\tilde{w}_{,\tilde{z}\tilde{\varsigma } \tilde{\varsigma }} \nonumber \\ & +\alpha a_{6}\tilde{T}\tilde{T}_{,\tilde{\varsigma }\tilde{\varsigma }\tilde{\varsigma }}+\alpha a_{7} \tilde{T}\tilde{w}_{,\tilde{z}\tilde{\varsigma }\tilde{\varsigma }\tilde{\varsigma }}+\alpha a_{8} \tilde{T}\tilde{T}_{,\tilde{\varsigma }\tilde{\varsigma }\tilde{\varsigma }\tilde{\varsigma }}+\alpha a_{9} \tilde{T}\tilde{w}_{,\tilde{z}\tilde{\varsigma }\tilde{\varsigma }\tilde{\varsigma }\tilde{\varsigma }}-\alpha a_{10}\tilde{T}_{,\tilde{z}}\tilde{T}_{,\tilde{z}\tilde{\varsigma }} \nonumber \\ & -\alpha \tilde{\tau }_{\theta }\left( \tilde{T}_{,\tilde{z}\tilde{\varsigma } }\right) ^{2}-\alpha \tilde{\tau }_{\theta }\tilde{T}_{,\tilde{\varsigma }}\tilde{T}_{, \tilde{z}\tilde{z}\tilde{\varsigma }}-\alpha \tilde{\tau }_{\theta }\tilde{T}_{,\tilde{ z}}\tilde{T}_{,\tilde{z}\tilde{\varsigma }\tilde{\varsigma }}-\alpha a_{2}\left( \tilde{T}_{, \tilde{z}}\right) ^{2}-\alpha a_{11}\tilde{T}_{,\tilde{\varsigma }}\tilde{T}_{,\tilde{ z}\tilde{z}} \nonumber \\ & +\alpha a_{6}\tilde{T}_{,\tilde{\varsigma }}\tilde{T}_{,\tilde{\varsigma }\tilde{\varsigma }}+\alpha a_{7}\tilde{T}_{,\tilde{\varsigma }}\tilde{w}_{,\tilde{z}\tilde{\varsigma }\tilde{\varsigma }}+\alpha a_{8}\left( \tilde{T}_{,\tilde{\varsigma }\tilde{\varsigma }}\right) ^{2}+\alpha a_{9}\tilde{T} _{,\tilde{\varsigma }\tilde{\varsigma }}\tilde{w}_{,\tilde{z}\tilde{\varsigma }\tilde{\varsigma }} \end{aligned}$$

20 $$\begin{aligned} \tilde{\sigma }=\tilde{w}_{,\tilde{z}}-a_{1}\tilde{T}-\alpha \tilde{T}\tilde{w }_{,\tilde{z}}+\alpha a_{1}\tilde{T}^{2}. \end{aligned}$$

The transformation to a moving wave frame is expressed as follows:

21 $$\begin{aligned} \tilde{w}\left( \tilde{z}, \tilde{\varsigma }\right) =\varphi \left( \epsilon \right) ,\quad \tilde{T}\left( \tilde{z}, \tilde{\varsigma }\right) =\psi \left( \epsilon \right) ,\quad \epsilon =\tilde{z} -\varrho \tilde{\varsigma }. \end{aligned}$$

Utilizing Eq. (21) in Eqs. (18), (19), and (20 ), we reveal that

22 $$\begin{aligned} A_{1}\varphi ^{^{\prime \prime }}+A_{2}\psi \varphi ^{^{\prime \prime }}-a_{1}\psi ^{^{\prime }}+\alpha \psi ^{^{\prime }}\varphi ^{^{\prime }}=0, \end{aligned}$$

23 $$\begin{aligned} 0= & A_{3}\varphi ^{^{(5)}}+A_{4}\varphi ^{^{(4)}}-A_{5}\varphi ^{^{(3)}}+A_{6}\psi ^{^{(4)}}+A_{7}\psi ^{^{(3)}}+A_{8}\psi ^{^{\prime \prime }}-A_{9}\psi \varphi ^{^{(5)}}-A_{10}\psi \varphi ^{^{(4)}} \nonumber \\ & +A_{11}\psi \varphi ^{^{(3)}}-A_{12}\psi \psi ^{^{(4)}}-A_{13}\psi \psi ^{^{(3)}}+A_{14}\psi \psi ^{^{\prime \prime }}+A_{15}\psi ^{^{\prime }}\psi ^{^{(3)}}+A_{16}\psi ^{^{\prime }}\psi ^{^{\prime \prime }} \nonumber \\ & +A_{10}\psi ^{^{\prime }}\varphi ^{^{(3)}}+A_{9}\psi ^{^{\prime \prime }}\varphi ^{^{(3)}}+A_{12}\left( \psi ^{^{\prime \prime }}\right) ^{2}+A_{17}\left( \psi ^{^{\prime }}\right) ^{2}, \end{aligned}$$

24 $$\begin{aligned} \tilde{\sigma }=\varphi ^{^{\prime }}-a_{1}\psi -\alpha \psi \varphi ^{^{\prime }}+\alpha a_{1}\psi ^{2}. \end{aligned}$$

where $$\varphi ^{^{(3)}}=\frac{d^{3}\varphi }{d\epsilon ^{3}},$$ $$A_{1}=\left( 1-\varrho ^{2}\right) ,$$ $$A_{2}=\alpha \left( \varrho ^{2}-1\right) ,A_{3}=-a_{9}\varrho ^{4},$$ $$A_{4}=a_{7}\varrho ^{3},$$

$$A_{5}=a_{5}\varrho ^{2},$$ $$A_{6}=\tilde{\tau }_{\theta }\varrho ^{2}-a_{8}\varrho ^{4},$$ $$A_{7}=a_{6}\varrho ^{3}-a_{3}\varrho ,A_{8}=a_{2}-a_{4}\varrho ^{2},$$

$$A_{9}=-\alpha A_{3},$$ $$A_{10}=-\alpha A_{4},$$ $$A_{11}=\alpha A_{5},$$ $$A_{12}=-\alpha A_{6},A_{13}=\alpha A_{7},$$ $$A_{14}=\alpha A_{8},$$

$$A_{15}=-2\alpha \tilde{\tau }_{\theta }\varrho ^{2},$$ $$A_{16}=\alpha \varrho \left( a_{10}+a_{11}-a_{6}\varrho ^{2}\right) ,$$ $$A_{17}=-\alpha a_{2}.$$

## Acquire exact solutions for the proposed model

Setting

25 $$\begin{aligned} \varphi =l\psi , \end{aligned}$$

in which *l* is constant.

Utilizing Eq. (25) in Eqs. (22) and (23), we get

26 $$\begin{aligned} A_{18}\psi ^{^{\prime \prime }}-a_{1}\psi ^{^{\prime }}+A_{19}\psi \psi ^{^{\prime \prime }}+A_{20}\left( \psi ^{^{\prime }}\right) ^{2}=0, \end{aligned}$$

27 $$\begin{aligned} 0= & A_{21}\psi ^{^{(5)}}+A_{22}\psi ^{^{(4)}}+A_{23}\psi ^{^{(3)}}+A_{8}\psi ^{^{\prime \prime }}-A_{24}\psi \psi ^{^{(5)}}-A_{25}\psi \psi ^{^{(4)}}+A_{26}\psi \psi ^{^{(3)}} \nonumber \\ & +A_{14}\psi \psi ^{^{\prime \prime }}+A_{16}\psi ^{^{\prime }}\psi ^{^{\prime \prime }}+A_{27}\psi ^{^{\prime }}\psi ^{^{(3)}}+A_{24}\psi ^{^{\prime \prime }}\psi ^{^{(3)}}+A_{12}\left( \psi ^{^{\prime \prime }}\right) ^{2}+A_{17}\left( \psi ^{^{\prime }}\right) ^{2}, \end{aligned}$$

where, $$A_{18}=lA_{1},$$ $$A_{19}=lA_{2},$$ $$A_{20}=\alpha l,$$ $$A_{21}=lA_{3},$$ $$A_{22}=A_{4}l+A_{6},$$ $$A_{23}=A_{7}-A_{5}l,$$

$$A_{24}=A_{9}l,$$ $$A_{25}=A_{12}+A_{10}l,$$ $$A_{26}=A_{11}l-A_{13},$$ $$A_{27}=A_{15}+A_{10}l.$$

Utilizing Eq. (26) in Eqs. (27), we get

28 $$\begin{aligned} 0= & A_{21}\psi ^{^{(5)}}+A_{22}\psi ^{^{(4)}}+A_{23}\psi ^{^{(3)}}+A_{28}\psi ^{^{\prime \prime }}+A_{29}\psi ^{^{\prime }}-A_{24}\psi \psi ^{^{(5)}}-A_{25}\psi \psi ^{^{(4)}} \nonumber \\ & +A_{26}\psi \psi ^{^{(3)}}+A_{30}\psi \psi ^{^{\prime \prime }}+A_{16}\psi ^{^{\prime }}\psi ^{^{\prime \prime }}+A_{27}\psi ^{^{\prime }}\psi ^{^{(3)}}+A_{24}\psi ^{^{\prime \prime }}\psi ^{^{(3)}}+A_{12}\left( \psi ^{^{\prime \prime }}\right) ^{2}, \end{aligned}$$

where, $$A_{28}=A_{8}-\frac{A_{17}A_{18}}{A_{20}},$$ $$A_{29}=\frac{a_{1}A_{17} }{A_{20}},$$ $$A_{30}=A_{14}-\frac{A_{17}A_{19}}{A_{20}}.$$

o apply the proposed method, it is necessary to determine the integer *N* By balancing the terms $\psi ^{(5)}$ and $(\psi ^{''})^2$, we find that $N=1$. Consequently, the solution to the resulting ODE can be expressed as follows:

29 $$\begin{aligned} \psi (\epsilon ) = s_0 + s_1 \Upsilon (\epsilon ) + \frac{s_2}{\Upsilon (\epsilon )}. \end{aligned}$$

Substituting Eq. (29) along with Eq. (5) into Eq. (28) and setting the coefficients of $\Upsilon (\epsilon )$ to zero, a system of nonlinear algebraic equations is derived. This system is solved using Mathematica software, leading to the following results.

**Case 1:** $$d_0=d_1=d_3=0$$

$$\begin{aligned} & s_1=0,~~d_2=-\frac{A_{30}}{A_{12}-A_{25}},~~A_{16}=-A_{26},\\ & A_{27}=0,~~s_0=\frac{-A_{12} A_{28}+A_{25} A_{28}+A_{22} A_{30}}{A_{12} A_{30}},~~A_{29}=-d_2 \left( -A_{24} d_2 s_0+A_{21} d_2+A_{26} s_0+A_{23}\right) ,\\ & s_2=\sqrt{\frac{A_{30}}{\left( A_{12}-A_{25}\right) d_4}}, \end{aligned}$$

Equation (28) is capable of yielding a hyperbolic solution.

30 $$\begin{aligned} & \tilde{T}=\cosh \left( \sqrt{-\frac{A_{30}}{A_{12}-A_{25}}}(\tilde{z} -\varrho \tilde{\varsigma })\right) +\frac{-A_{12} A_{28}+A_{25} A_{28}+A_{22} A_{30}}{A_{12} A_{30}}, \end{aligned}$$

31 $$\begin{aligned} & \tilde{w}=l \bigg (\cosh \left( \sqrt{-\frac{A_{30}}{A_{12}-A_{25}}}(\tilde{z} -\varrho \tilde{\varsigma })\right) +\frac{-A_{12} A_{28}+A_{25} A_{28}+A_{22} A_{30}}{A_{12} A_{30}}\bigg ), \end{aligned}$$

and the stress tensor component will be :

32 $$\begin{aligned} & \tilde{\sigma }=\frac{1}{A_{12}^2 A_{30}^2} \left( - A_{12} \left( \alpha A_{28} + (1 - \alpha \cosh \left[ (\tilde{z} -\varrho \tilde{\varsigma }) \sqrt{-\frac{A_{30}}{A_{12} - A_{25}}} \right] ) A_{30} \right) + \alpha (A_{25} A_{28} + A_{22} A_{30}) \right) \times \nonumber \\ & \bigg ( -l \sinh \left[ (\tilde{z} -\varrho \tilde{\varsigma }) \sqrt{-\frac{A_{30}}{A_{12} - A_{25}}} \right] A_{12} A_{30} \sqrt{-\frac{A_{30}}{A_{12} - A_{25}}} \nonumber \\ & + a_{1} \left( A_{25} A_{28} + A_{22} A_{30} + A_{12} (-A_{28} + \cosh \left[ (\tilde{z} -\varrho \tilde{\varsigma }) \sqrt{\frac{A_{30}}{-A_{12} + A_{25}}} \right] A_{30}) \right) \bigg ) \end{aligned}$$

**Case 2:** $$d_2=d_4=0$$

$$\begin{aligned} s_{2} = & 0,~A_{{12}} = 2A_{{25}} ,~A_{{21}} = \frac{{ - A_{{16}} s_{1} - 2A_{{26}} s_{1} }}{{15d_{3} }},~A_{{22}} = A_{{25}} s_{0} ,~A_{{23}} \\ = & - A_{{26}} s_{0} ,~A_{{24}} = 0,~A_{{27}} = A_{{25}} ,~A_{{28}} = - A_{{25}} d_{1} s_{1} , \\ d_{1} = & \frac{{5A_{{29}} }}{{\left( {3A_{{26}} - A_{{16}} } \right)s_{1} }},~A_{{30}} = 0. \\ \end{aligned}$$

Equation (28) has the potential to produce Weierstrass elliptic solution

33 $$\begin{aligned} \tilde{T}= s_1 \wp \left( \frac{1}{2}(\tilde{z} -\varrho \tilde{\varsigma }) \sqrt{d_3};-\frac{4 d_1}{d_3},-\frac{4 d_0}{d_3}\right) +\dfrac{A_{22}}{A_{25}}. \end{aligned}$$

34 $$\begin{aligned} \tilde{w} = l \left( s_1 \wp \left( \frac{1}{2}(\tilde{z} - \varrho \tilde{\varsigma }) \sqrt{d_3};\ -\frac{4 d_1}{d_3},\ -\frac{4 d_0}{d_3} \right) + \frac{A_{22}}{A_{25}} \right) \end{aligned}$$

and the stress tensor component is :

35 $$\begin{aligned} \tilde{\sigma } =&\frac{1}{30 A_{25}^2} \Bigg ( \left( \alpha A_{22} + A_{25} \left( \alpha s_1 \, \wp \left( \frac{(\tilde{z} -\varrho \tilde{\varsigma }) \sqrt{ -\frac{(A_{16} + 2A_{26}) s_1}{A_{21}} }}{2 \sqrt{15}}; \frac{60 A_{21} d_1}{(A_{16} + 2A_{26}) s_1}, \frac{60 A_{21} d_0}{(A_{16} + 2A_{26}) s_1} \right) - 1 \right) \right) \nonumber \\&\quad \times \left( 30 a_1 \left( A_{25} s_1 \, \wp \left( \frac{(\tilde{z}-\varrho \tilde{\varsigma }) \sqrt{ -\frac{(A_{16} + 2A_{26}) s_1}{A_{21}} }}{2 \sqrt{15}}; \frac{60 A_{21} d_1}{(A_{16} + 2A_{26}) s_1}, \frac{60 A_{21} d_0}{(A_{16} + 2A_{26}) s_1} \right) + A_{22} \right) \nonumber \right. \\&\left. \qquad - \sqrt{15} A_{25} l s_1 \sqrt{ -\frac{(A_{16} + 2A_{26}) s_1}{A_{21}} } \, \wp '\left( \frac{(\tilde{z} -\varrho \tilde{\varsigma }) \sqrt{ -\frac{(A_{16} + 2A_{26}) s_1}{A_{21}} }}{2 \sqrt{15}}; \frac{60 A_{21} d_1}{(A_{16} + 2A_{26}) s_1}, \frac{60 A_{21} d_0}{(A_{16} + 2A_{26}) s_1} \right) \right) \Bigg ) \end{aligned}$$

**Case 3:** $$d_0=d_1=d_2=0$$

$$\begin{aligned} & s_1=0,~d_3=-\frac{2 A_{28}}{A_{18} s_2},~A_{29}=-\frac{1}{2} A_{16} d_3 s_2,~A_{30}=0,~~s_2=\dfrac{1}{\sqrt{-d_2}}, \end{aligned}$$

Equation (28) has the potential to produce exponential solution

36 $$\begin{aligned} \tilde{T}= s_0-\frac{A_{18} e^{\frac{A_{28} (\tilde{z} -\varrho \tilde{\varsigma })}{A_{18}}}}{A_{28}}. \end{aligned}$$

37 $$\begin{aligned} \tilde{w}= l\left( s_0-\frac{A_{18} e^{\frac{A_{28} (\tilde{z} -\varrho \tilde{\varsigma })}{A_{18}}}}{A_{28}}\right) . \end{aligned}$$

and the stress tensor component will be :

38 $$\begin{aligned} \tilde{\sigma }=\frac{\left( A_{28} \left( \alpha s_0-1\right) -\alpha A_{18} e^{\frac{A_{28} (\tilde{z} -\varrho \tilde{\varsigma })}{A_{18}}}\right) \left( a_1 \left( A_{28} s_0-A_{18} e^{\frac{A_{28} (z-\nu t)}{A_{18}}}\right) +A_{28} l e^{\frac{A_{28} (z-\nu t)}{A_{18}}}\right) }{A_{28}^2}. \end{aligned}$$

**Case 4:** $$d_0=d_1=d_4=0$$

$$\begin{aligned} & s_2=0,~s_1=-\frac{15 A_{21} d_3}{A_{16}+2 A_{26}},~A_{22}=\frac{12 A_{25} d_3 s_0-3 A_{12} d_2 s_1+2 A_{25} d_2 s_1}{12 d_3},~A_{23}=-\frac{A_{26} \left( 3 d_3 s_0-d_2 s_1\right) }{3 d_3}, \\ & A_{24}=0,~~A_{27}=\frac{1}{4} \left( 10 A_{25}-3 A_{12}\right) ,~A_{28}=-\frac{d_2 \left( 15 A_{12} d_3 s_0-30 A_{25} d_3 s_0-3 A_{12} d_2 s_1+2 A_{25} d_2 s_1\right) }{12 d_3},\\ & A_{29}=-\frac{\left( 3 A_{26}-A_{16}\right) d_2^2 s_1}{15 d_3},~d_2=\frac{4 A_{30}}{5 \left( A_{12}-2 A_{25}\right) }, \end{aligned}$$

Eq. (28) has the potential to produce bell shaped solitary solution

39 $$\begin{aligned} \tilde{T}= \frac{12 A_{21} A_{30} \text {sech}^2\left( \sqrt{\frac{A_{30}}{5 \left( A_{12}-2 A_{25}\right) }} (\tilde{z} -\varrho \tilde{\varsigma })\right) }{\left( A_{12}-2 A_{25}\right) \left( A_{16}+2 A_{26}\right) }+s_0. \end{aligned}$$

40 $$\begin{aligned} \tilde{w}= l\left( \frac{12 A_{21} A_{30} \text {sech}^2\left( \sqrt{\frac{A_{30}}{5 \left( A_{12}-2 A_{25}\right) }} (\tilde{z} -\varrho \tilde{\varsigma })\right) }{\left( A_{12}-2 A_{25}\right) \left( A_{16}+2 A_{26}\right) }+s_0\right) . \end{aligned}$$

and the stress tensor component will be :

41 $$\begin{aligned} \tilde{\sigma }=a_1 \left( \frac{12 A_{21} A_{30} \text {sech}^2\left( \sqrt{\frac{A_{30}}{5 A_{12}-10 A_{25}}} (\tilde{z} -\varrho \tilde{\varsigma })\right) }{\left( A_{12}-2 A_{25}\right) \left( A_{16}+2 A_{26}\right) }+s_0\right) \left( \frac{12 \alpha A_{21} A_{30} \text {sech}^2\left( \sqrt{\frac{A_{30}}{5 A_{12}-10 A_{25}}} (\tilde{z} -\varrho \tilde{\varsigma })\right) }{\left( A_{12}-2 A_{25}\right) \left( A_{16}+2 A_{26}\right) }+\alpha s_0-1\right) . \end{aligned}$$

**Case 5:** $$d_0=d_3=d_4=0$$

$$\begin{aligned} & d_2=-\frac{4 A_{30}}{5 \left( 2 A_{25}-A_{12}\right) },~ s_1=0,~s_2=\frac{15 A_{21} d_1}{-A_{16}-2 A_{26}},~A_{22}=\frac{12 A_{25} d_1 s_0-3 A_{12} d_2 s_2+2 A_{25} d_2 s_2}{12 d_1}, \nonumber \\ & ~A_{23}=\frac{A_{26} d_2 s_2-3 A_{26} d_1 s_0}{3 d_1},~A_{24}=0;A_{27}=\frac{1}{4} \left( 10 A_{25}-3 A_{12}\right) ,\nonumber \\ & ~A_{28}=\frac{d_2 \left( -15 A_{12} d_1 s_0+30 A_{25} d_1 s_0+3 A_{12} d_2 s_2-2 A_{25} d_2 s_2\right) }{12 d_1},~A_{29}=-\frac{\left( 3 A_{26}-A_{16}\right) d_2^2 s_2}{15 d_1}, \end{aligned}$$

Eq. (28) has the potential to produce sigular hyperbolic solution

42 $$\begin{aligned} \tilde{T}=\frac{15 A_{21}}{\left( -A_{16}-2 A_{26}\right) \left( \frac{5 \left( 2 A_{25}-A_{12}\right) }{8 A_{30}}-\frac{5 \left( 2 A_{25}-A_{12}\right) \sinh \left( \frac{4 \sqrt{-\frac{A_{30}}{2 A_{25}-A_{12}}}(\tilde{z} -\varrho \tilde{\varsigma })}{\sqrt{5}}\right) }{8 A_{30}}\right) }+s_0 \end{aligned}$$

43 $$\begin{aligned} \tilde{w}=l (\frac{15 A_{21}}{\left( -A_{16}-2 A_{26}\right) \left( \frac{5 \left( 2 A_{25}-A_{12}\right) }{8 A_{30}}-\frac{5 \left( 2 A_{25}-A_{12}\right) \sinh \left( \frac{4 \sqrt{-\frac{A_{30}}{2 A_{25}-A_{12}}} (\tilde{z} -\varrho \tilde{\varsigma })}{\sqrt{5}}\right) }{8 A_{30}}\right) }+s_0). \end{aligned}$$

and the stress tensor component will be :

44 $$\begin{aligned} & \tilde{\sigma } = \left[ \left( A_{12} - 2 A_{25}\right) \left( A_{16} + 2 A_{26}\right) \left( \alpha s_0 - 1\right) \left( \sinh \left( 4 \sqrt{\frac{A_{30}}{5 A_{12} - 10 A_{25}}} (\tilde{z} -\varrho \tilde{\varsigma })\right) - 1\right) - 24 \alpha A_{21} A_{30} \right] \times \nonumber \\ & \quad \bigg [ 10 a_1 \left( \sinh \left( 4 \sqrt{\frac{A_{30}}{5 A_{12} - 10 A_{25}}}(\tilde{z} -\varrho \tilde{\varsigma })\right) - 1\right) \times \nonumber \\ & \quad \quad \bigg ( \left( A_{12} - 2 A_{25}\right) \left( A_{16} + 2 A_{26}\right) s_0 \left( \sinh \left( 4 \sqrt{\frac{A_{30}}{5 A_{12} - 10 A_{25}}}(\tilde{z} -\varrho \tilde{\varsigma })\right) - 1\right) \nonumber \\ & \quad \quad \quad - 24 A_{21} A_{30} - 192 \sqrt{5} A_{21} A_{30} \sqrt{\frac{A_{30}}{A_{12} - 2 A_{25}}} \, l \cosh \left( 4 \sqrt{\frac{A_{30}}{5 A_{12} - 10 A_{25}}}(\tilde{z} -\varrho \tilde{\varsigma })\right) \bigg ) \bigg ] \nonumber \\ & \quad \times \dfrac{1}{10 \left( A_{12} - 2 A_{25}\right) ^2 \left( A_{16} + 2 A_{26}\right) ^2 \left( \sinh \left( 4 \sqrt{\frac{A_{30}}{5 A_{12} - 10 A_{25}}}(\tilde{z} -\varrho \tilde{\varsigma })\right) - 1\right) ^3} \end{aligned}$$

## Discussion

This part of the study provides detailed graphical illustrations of specific solutions to the given problem in both two-dimensional and three-dimensional formats. These visualizations enhance the understanding of the behavior and characteristics of the solutions. Copper is selected as the thermoelastic material for this analysis due to its notable physical properties, which make it an appropriate choice for the investigation. The necessary physical constants for copper are assigned accurate values, as outlined in the following^59^:

$$\begin{aligned} \mu _{0} = & 38.6 \times 10^{9} \;{\text{N}}\;{\text{m}}^{{ - 2}} ,\;\;c_{e} = 3.831 \times 10^{2} \;{\text{J}}\;{\text{kg}}^{{ - 1}} .K^{{ - 1}} ,\;\;k_{0} = 100\;{\text{w}}\;{\text{m}}^{{ - 1}} .K^{{ - 1}} , \\ T_{0} = & {\text{ }}2.93 \times 10^{2} {\text{K}},\;\;\rho _{0} = 89.54 \times 10^{2} \;{\text{kg}}\;{\text{m}}^{{ - 3}} ,\;\;\lambda _{0} = 77.6 \times 10^{9} \;{\text{N}}\;{\text{m}}^{{ - 2}} , \\ \alpha _{t} = & 1.78 \times 10^{{ - 5}} {\text{K}}^{{ - 1}} ,\;\;k_{0}^{*} = 368\;\;{\text{w}}\;{\text{m}}^{{ - 1}} \;{\text{K}}^{{ - 1}} \;{\text{s}}^{{ - 1}} . \\ \end{aligned}$$

Parameter range and stability consideration:

To ensure the stability and physical validity of the obtained analytical solutions, we carefully selected the material and model parameters within realistic ranges that reflect temperature-sensitive thermoelastic media. The ranges used in our simulations and analysis are summarized below:

1. Phase-lag of heat flux ($\tau _q$): $0.1\preceq \tau _q \preceq 1$
2. Phase-lag of temperature gradient ($\tau _{\theta }$): $0.05\preceq \tau _{\theta } \preceq 0.8$
3. Phase lag of thermal displacement gradient ($\tau _{\nu }$): $0.01\preceq \tau _{\nu } \preceq 0.5$
4. Temperature-sensitivity parameter ($\alpha$): $1\preceq \alpha \preceq 10$ covering a wide range from temperature-independent to strongly temperature-dependent materials
5. Thermal conductivity (*k*), specific heat ($c_e$), and density ($\rho$): Selected based on standard values for copper and similar engineering materials

**Fig. 1:**
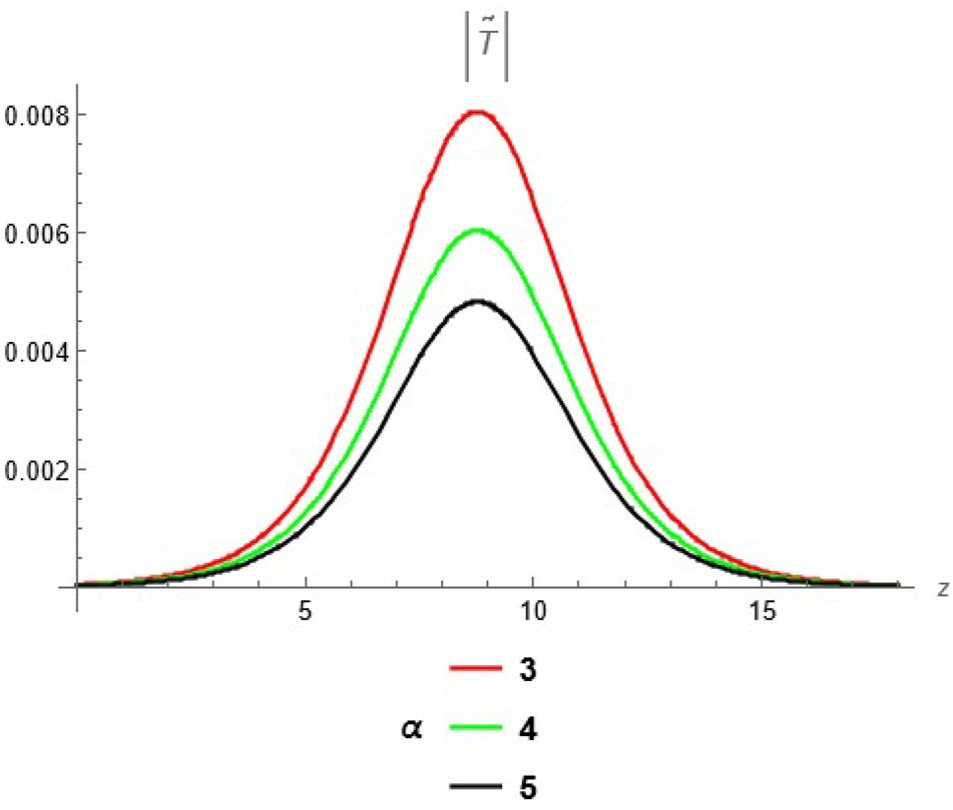
Bright soliton solution at different values of $\alpha$.

**Fig. 2:**
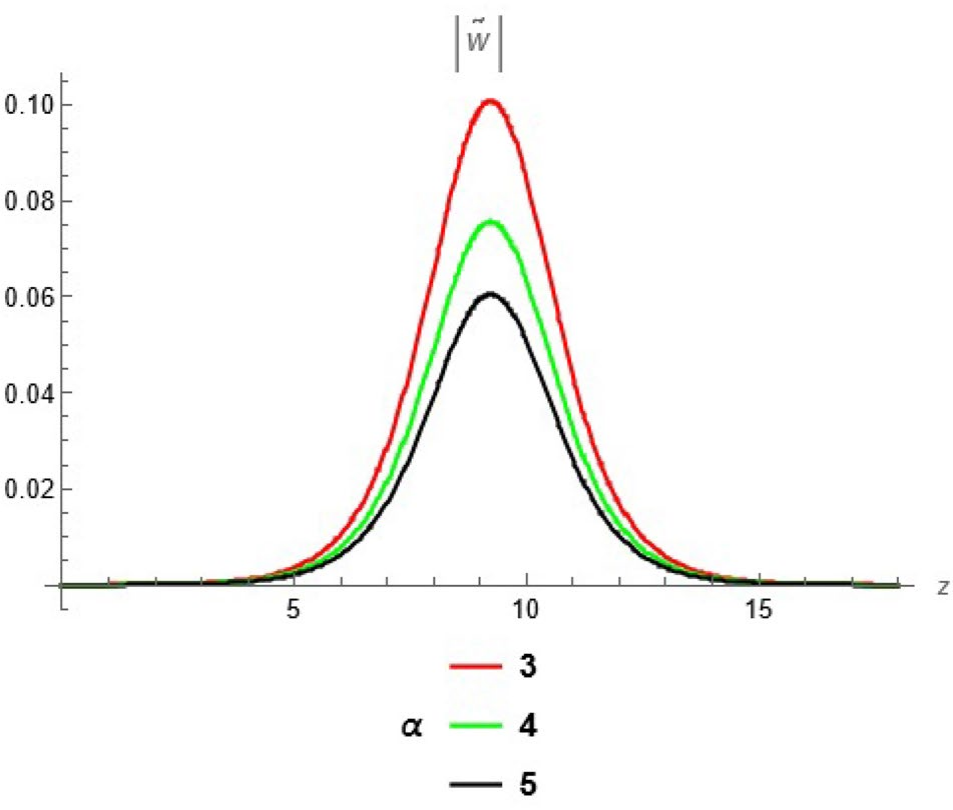
Bright solution for displacement at different values of $\alpha$.

**Fig. 3:**
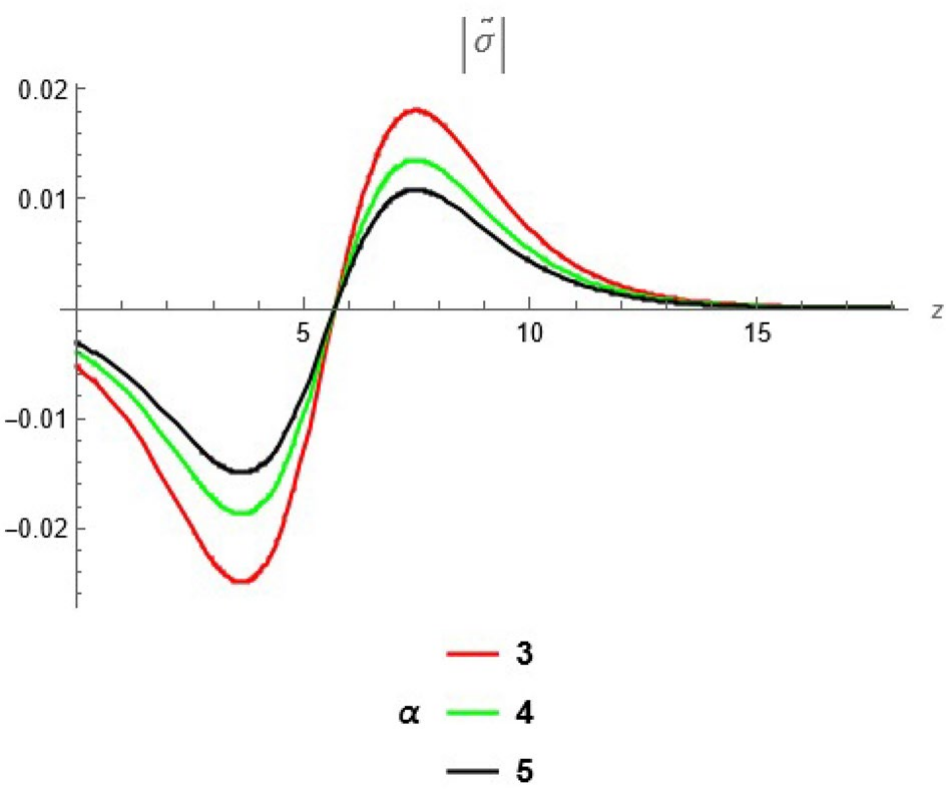
solution for stress tensor at different values of $\alpha$.

**Fig. 4:**
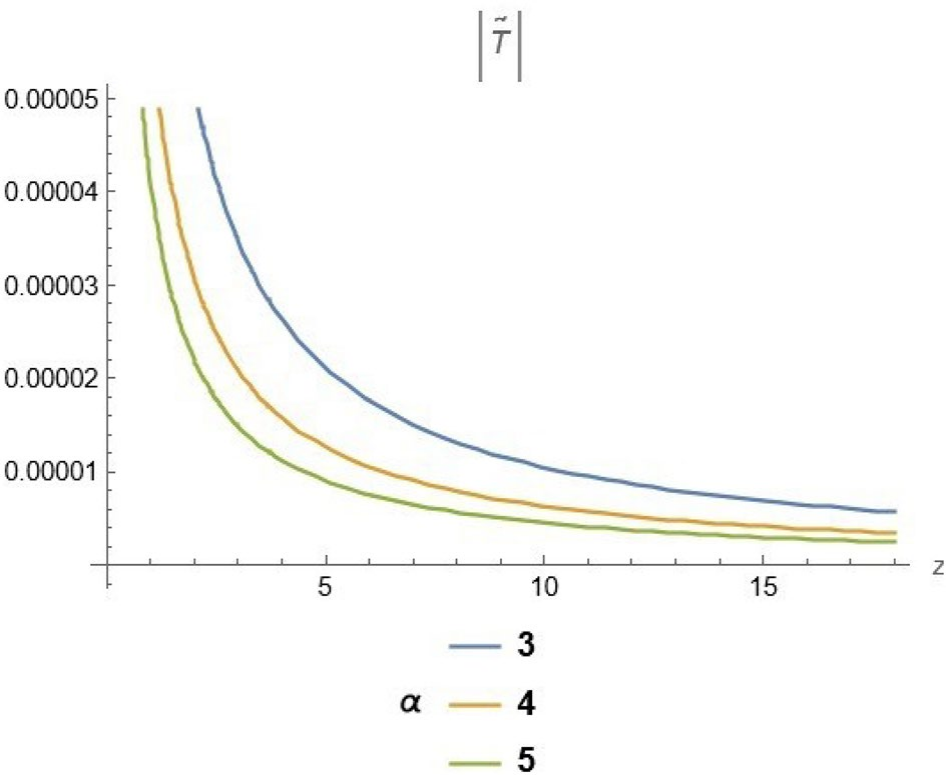
solution for temperature at different values of $\alpha$.

**Fig. 5:**
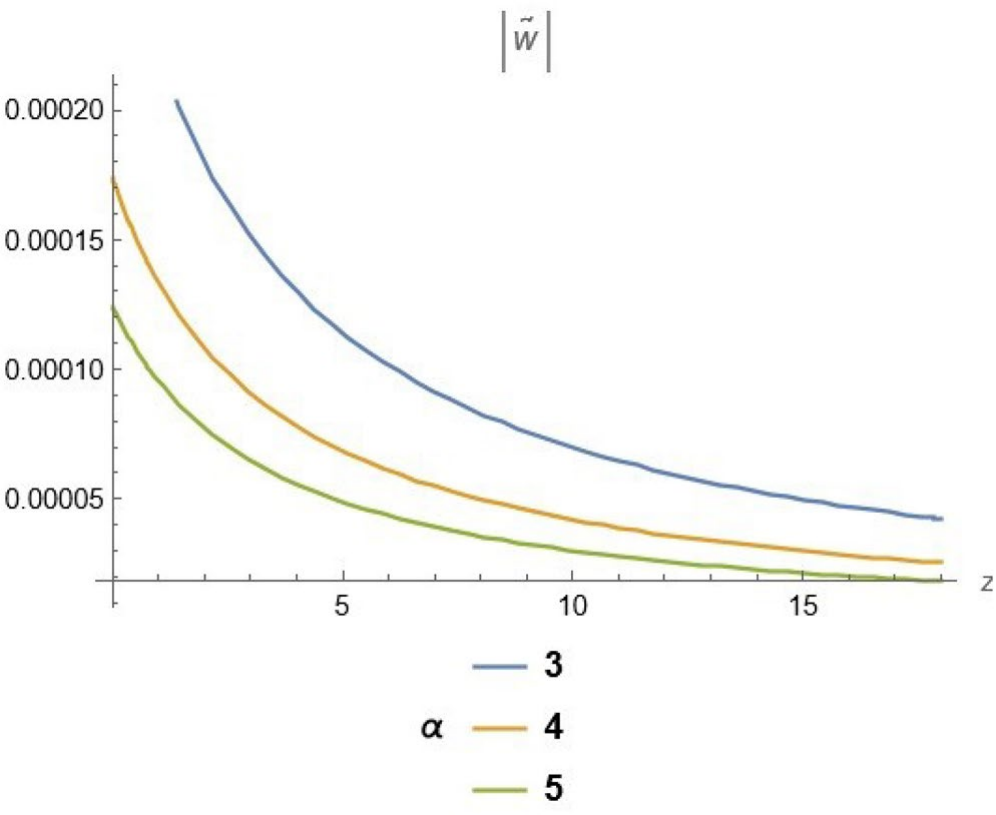
solution for displacement at different values of $\alpha$.

**Fig. 6:**
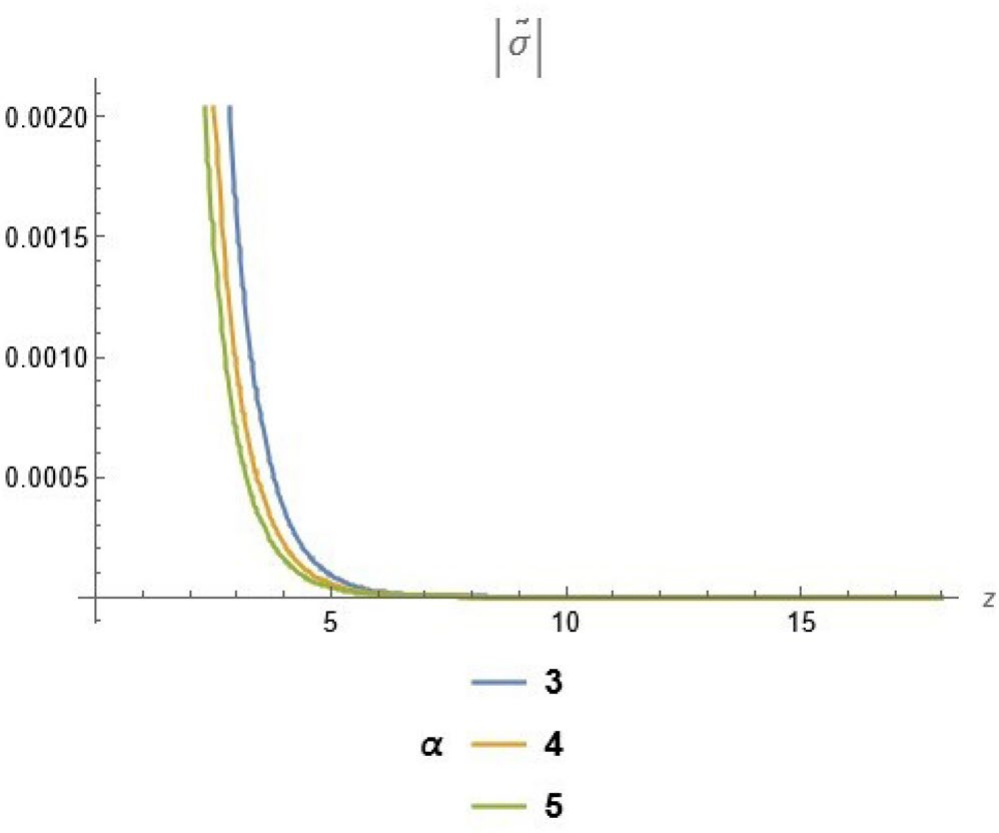
solution for stress tensor at different values of $\alpha$.

**Fig. 7:**
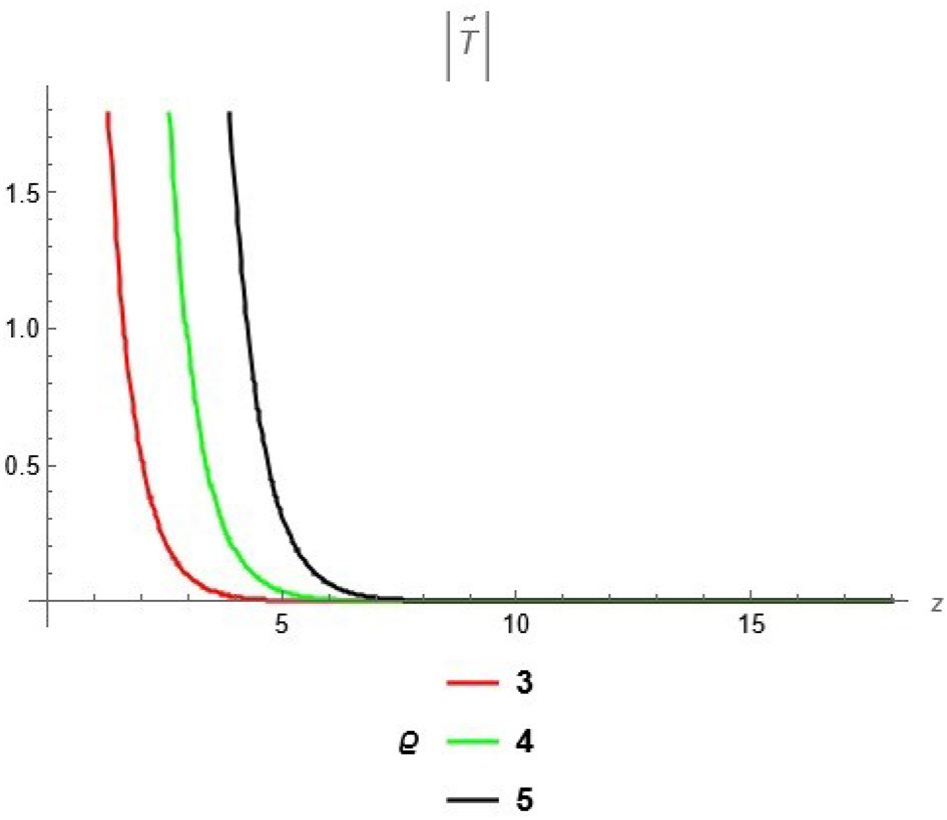
solution for temperature (36) at different values of $\varrho$.

**Fig. 8:**
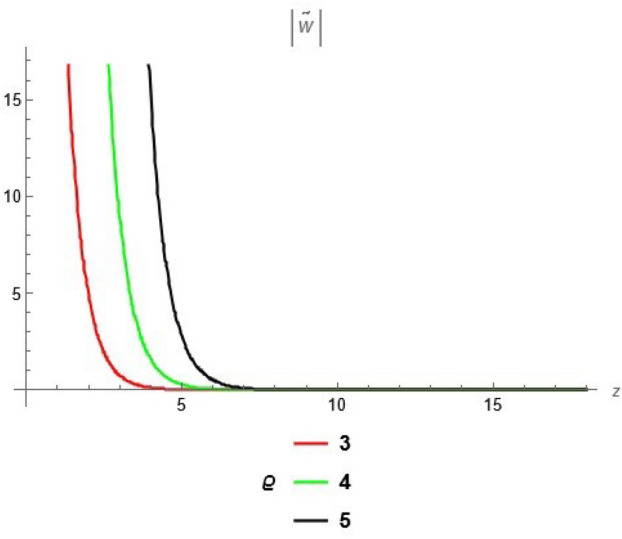
solution for displacement (37) at different values of $\varrho$.

**Fig. 9:**
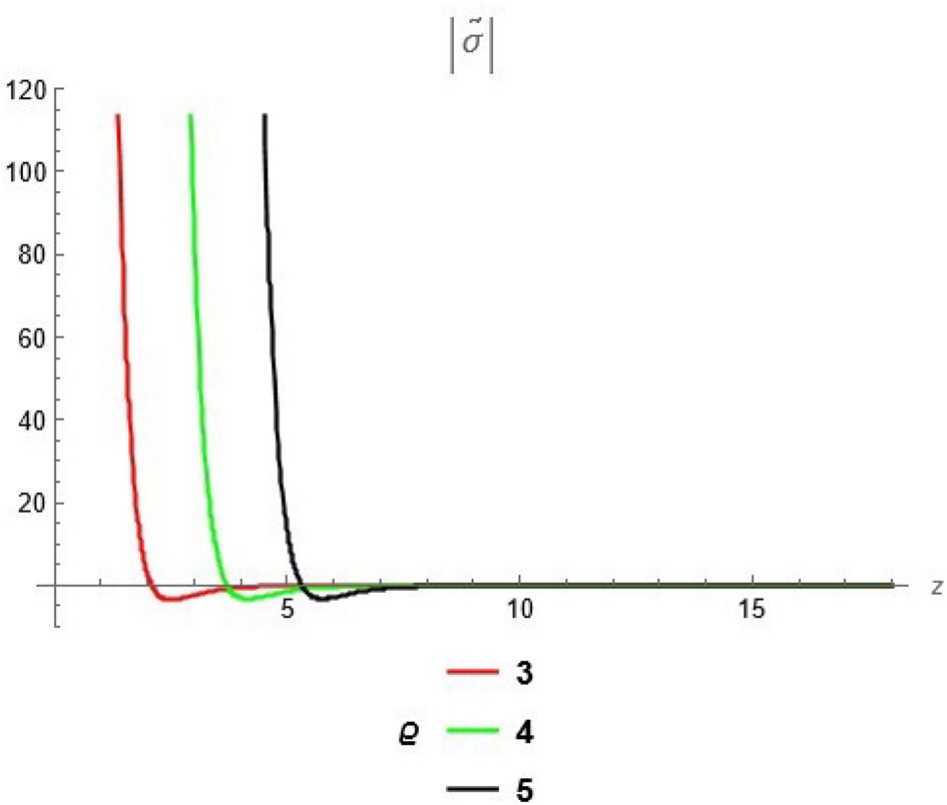
solution for tensor stress (38) at different values of $\varrho$.

To emphasize the features of the obtained results, selected solutions are displayed through graphical simulations. Figure (1) shows the graphical representation of a bright soliton solution to the Eq. (39) at different values of $\alpha$ with $s_0=0,~~\varrho =2.71,~~\tau _{q}=0.63,~~t=3.5$. This figure displays the magnitude of the temperature field $\tilde{T}$ along the spatial coordinate *z*. It shows a bell-shaped profile for each value of $\alpha$. The value of $\tilde{T}$ decreases as $\alpha$ increases. This suggests that when the material properties are more sensitive to temperature, the resulting thermal distribution becomes less intense and more localized. Physically, this could mean that the material better regulates or diffuses the thermal wave when the temperature dependence is higher, leading to reduced thermal spikes. Figure (2) presents a graphical simulation of a bright solution for Eq. (40) at different values of $\alpha$ with $s_0=0,~~\varrho =4.4,~~l=10,~~t=2.25$. It is clear that the displacement reaches a peak near the center (around $z=10$) and decays symmetrically away from it, showing a localized wave behavior. As $\alpha$ increases, the displacement value decreases, indicating that a stronger temperature dependence tends to suppress the mechanical response. Figure (3) illustrates a graphical simulation of a solution for Eq. (41) at different values of $\alpha$ with $s_{0} = 0,~\;{\varrho } = 3.5,~\;\tau _{q} = 0.51,~\;l = - 10,\;~t = 1.55$. The value of stress increases as $\alpha$ increases in the spatial range $~~z\in [0,5]$, while the opposite trend is observed in the range $z\in [5,15]$. This suggests that when the material properties exhibit stronger temperature dependence (i.e., larger $\alpha$), the stress response becomes more localized near the source region and decays more rapidly further away. Physically, this could mean that higher temperature dependence enhances the initial thermo-mechanical coupling, resulting in greater stress generation near the thermal excitation zone. However, as the thermal wave propagates through the medium, the stronger coupling also facilitates faster energy dispersion, leading to reduced stress levels at larger distances. This dual behavior reflects the complex interplay between heat conduction and elastic deformation in temperature-sensitive materials. Figure (4) clarifies a graphical simulation of a solution for Eq. (42) at different values of $\alpha$ with $s_0=0,~~\varrho =4.1,~~\tau _{q}=0.63,~~t=2.25$. Distribution of $\tilde{T}$ Temperature Profile (Second Case or Asymptotic Behavior) Unlike the previous figures, this plot represents a monotonically decreasing temperature profile over space. As $\alpha$ increases (from blue to yellow to green), the decay becomes steeper, and the thermal magnitude decreases more rapidly with distance. This implies that in materials with higher temperature sensitivity, the thermal energy dissipates faster, and the heat flux diminishes more rapidly from the source region. The observed trend is consistent with a thermally conductive medium becoming more effective at diffusing heat when its thermal conductivity depends strongly on temperature. Figure (5) demonstrates a graphical simulation of a solution for Eq. (43) at different values of $\alpha$ with $s_0=0,~~\varrho =4.1,~~\tau _{q}=0.63,~~l=10,~~t=2.25$. As $\alpha$ increases, the displacement value decreases, indicating that a stronger temperature dependence tends to suppress the mechanical response. Figure (6) shows a graphical simulation of a solution for Eq. (44) at different values of $\alpha$ with $s_0=0,~~\varrho =3.21,~~\tau _{q}=0.51,~~l=36,~~t=0.1$. As $\alpha$ decreases, the stress tensor value increases, indicating that a stronger temperature dependence tends to amplify the internal mechanical stresses within the material. This suggests that when the material properties are more sensitive to temperature variations (i.e., lower $\alpha$), thermal fluctuations have a more pronounced effect on the stress distribution, possibly due to intensified thermal gradients or delayed thermal relaxation. Figure (7) shows a graphical simulation of a solution for Eq. (36) at different values of $\varrho$ with $s_0=0,~~l=10,~~\omega =-44.6,~~\gamma _{0}=-15.2,~~t=0.65$. As wave speed increases, the thermal wave travels faster, leading to faster and more concentrated delivery of energy. This results in elevated temperature values due to the reduced time for thermal diffusion, enhanced thermomechanical coupling, and non-equilibrium heat conduction effects, all of which contribute to a more intense thermal response in the medium. Figure (8) shows a graphical simulation of a solution for Eq. (37) at different values of $\varrho$ with $s_0=0,~~l=10,~~\omega =-44.6,~~\gamma _{0}=-15.2,~~t=0.65,~~\alpha =1$. As wave speed increases, the displacement values rise due to more efficient energy transfer, reduced dispersion, and enhanced mechanical response. This reflects a medium where disturbances travel quickly, causing stronger deformations over shorter time intervals. The increased wave speed intensifies the dynamic interaction between stress and strain, resulting in higher peak displacements and more localized mechanical effects. Figure (9) shows a graphical simulation of a solution for Eq. (38) at different values of $\varrho$ with $s_0=0,~~l=10,~~\omega =-44.2,~~\gamma _{0}=-17.6,~~t=0.8$. As wave speed increases, the stress tensor values rise due to faster and more concentrated momentum transfer, stiffer material response, and stronger coupling between thermal and mechanical fields. The increased wave speed leads to higher internal force densities, reduced energy dispersion, and sharper stress peaks, reflecting more intense and localized mechanical interactions within the material. Validation of Results with Existing Literature:

To validate the accuracy of the present analytical solutions, we compared our findings with previously published results in similar thermoelastic frameworks. In particular: When the temperature dependence parameter $\alpha \rightarrow 0$ and the third phase-lag parameter ($\tau _{\nu }$) are neglected, our model reduces to a special case of the Dual-Phase-Lag model as studied by Othman et al.^60^. Under the limiting conditions, our wave propagation and attenuation profiles closely match the results reported in their work. Similarly, when all phase-lag parameters are set to zero, our solution aligns with classical thermoelasticity, showing good agreement with the results presented in^61^.

## Conclusion

The IMETF method has established itself as a powerful and flexible analytical tool for exploring temperature-dependent behavior in thermoelastic materials, particularly within the framework of the 3PHL model. Its capacity to produce an extensive array of precise solutions. These include hyperbolic, singular hyperbolic, exponential, Weierstrass elliptic, and bell-shaped solitary wave solutions. Its strength and adaptability in solving complex mathematical models. This method facilitates theoretical derivations and offers practical insights via graphical simulations. Visual representations of displacement profiles, heat fields, and stress components are essential to corroborate theoretical results. These graphical results provide essential insights for researchers and practitioners, facilitating the interpretation of thermoelastic materials’ responses to various thermal and mechanical stimuli.

The accuracy of the derived analytical wave solutions was validated through comparison with previously published results under special conditions. Our results showed excellent agreement with the dual-phase-lag^60^ and classical thermoelasticity^61^ models when the corresponding parameters were appropriately limited. This consistency confirms the validity of the present model and demonstrates its ability to generalize and extend classical theories while capturing additional physical effects such as temperature dependence and three-phase-lag heat conduction.

Moreover, the IMETF methodology substantially enhances the theoretical comprehension of thermoelastic systems. It creates a comprehensive analytical framework that can be applied in future research, both in academic research and engineering applications. By elucidating the complex interactions between temperature and deformation in these materials, this method opens new avenues for innovation. It holds great promise in enhancing the design and optimization of thermoelastic devices and systems, thereby playing a pivotal role in the advancement of emerging technologies and interdisciplinary methodologies.

## Data Availability

The datasets used and/or analysed during the current study are available from the corresponding author on reasonable request.

## List of symbols

*w*: Component of the displacement vector
*k*: Thermal conductivity
$\lambda$ and $\mu$: Lame’s constants
$$k^{*}$$: Additional material
$\sigma$: Component of the stress tensor
$\alpha _{t}$: Linear thermal expansion coefficient, where $\gamma =\left( 3\lambda +2\mu \right) \alpha _{t}$
*T*: Temperature
$$T_{0}$$: Reference temperature
$c_{e}$: Specific heat at the constant strain
$\tau _{\theta }$: Phase-lag of the temperature gradient
$\rho$: Density of material
$\tau _{\nu }$: Phase lag of thermal displacement gradient
$$\varsigma$$: Time
$\tau _{q}$: Phase-lag of the heat flux
